# Fatal Rapidly Progressive Interstitial Lung Disease in Anti-Melanoma Differentiation-Associated Gene 5 (MDA5) Dermatomyositis in a Brazilian Patient: A Case Report

**DOI:** 10.7759/cureus.111496

**Published:** 2026-06-25

**Authors:** Izabella Zgoda, Amanda A Machado, Gabrielle C Silva, Rafael Y Kaiya, Laura P Vasconcelos, Vitor L Dias

**Affiliations:** 1 Internal Medicine, Hospital Universitário Cajuru, Curitiba, BRA; 2 Internal Medicine, Pontifícia Universidade Católica do Paraná, Curitiba, BRA; 3 Respiratory Medicine, Hospital Universitário Cajuru, Curitiba, BRA

**Keywords:** anti-melanoma differentiation-associated gene 5, dermatomyositis, idiopathic inflammatory myopathy, interstitial lung disease, pneumomediastinum

## Abstract

Anti-melanoma differentiation-associated gene 5 (anti-MDA5) dermatomyositis (DM) is a subtype of idiopathic inflammatory myopathy. It is characterized by prominent cutaneous manifestations, minimal muscle involvement, and a strong association with interstitial lung disease (ILD), particularly rapidly progressive ILD (RP-ILD), a life-threatening complication with high early mortality. Although RP-ILD is less frequently reported in Western populations than in Asian cohorts, severe presentations may still occur. We report the case of a previously healthy 35-year-old woman who presented with typical DM features and progressive dyspnea. Chest computed tomography (CT) revealed an organizing pneumonia pattern consistent with ILD. Despite early aggressive immunosuppression, the patient developed pneumomediastinum, pneumothorax, and worsening diffuse ground-glass opacities. Anti-MDA5 positivity was confirmed, and even after rescue therapy and intensive supportive care, the patient died. This case illustrates the fulminant course of anti-MDA5 DM with RP-ILD and severe structural complications, emphasizing the need for early recognition and aggressive immunosuppression even in populations with a lower reported prevalence of RP-ILD.

## Introduction

Dermatomyositis (DM) associated with the anti-melanoma differentiation-associated gene 5 (anti-MDA5) antibody is a specific subtype of idiopathic inflammatory myopathies, characterized by an exuberant cutaneous phenotype, arthralgia, and absent or mild muscle weakness and/or elevated muscle enzyme levels [1-5]. Anti-MDA5 is a myositis-specific autoantibody directed against MDA5, a cytosolic RNA sensor involved in type I interferon-mediated antiviral immunity. Its pathogenic role is associated with excessive type I interferon production, resulting in a hyperinterferonemic state that triggers autoinflammatory immune responses [1]. The presence of anti-MDA5 antibodies is associated with a substantially increased risk of interstitial lung disease (ILD), especially rapidly progressive ILD (RP-ILD). As the leading determinant of prognosis in this patient population, RP-ILD continues to be associated with significant mortality despite aggressive immunosuppressive therapies [1-7].

In Brazil, recent studies suggest that patients with anti-MDA5 DM have a distinct phenotype from that described in Asian cohorts, with a lower prevalence of ILD and RP-ILD [8]. In this context, we report the case of a young female patient with anti-MDA5 DM, RP-ILD, and extensive pneumomediastinum, with a fatal outcome despite early combined immunosuppression, illustrating an aggressive variant of the disease in a population in which RP-ILD is less frequently described.

## Case presentation

A 35-year-old female patient, previously healthy, was admitted in June 2025 due to acute respiratory failure. There was no history of recent vaccination or documented infection preceding symptom onset. Three months before admission, she developed generalized skin lesions (Figure 1), which included typical DM skin manifestations, such as Gottron papules, an erythematous V-sign rash over the anterior neck and chest, heliotrope, and palmar thickening. During the same period, she also complained of arthralgia, stiffness, and edema of the hand, wrist, and knee joints bilaterally, as well as mild proximal muscle weakness. She subsequently developed progressively worsening dyspnea and required a prior hospitalization with a presumptive diagnosis of pneumonia. However, her respiratory symptoms and pulmonary abnormalities did not improve despite broad-spectrum antibiotic therapy, prompting further investigation and the current hospital admission.

**Figure 1:**
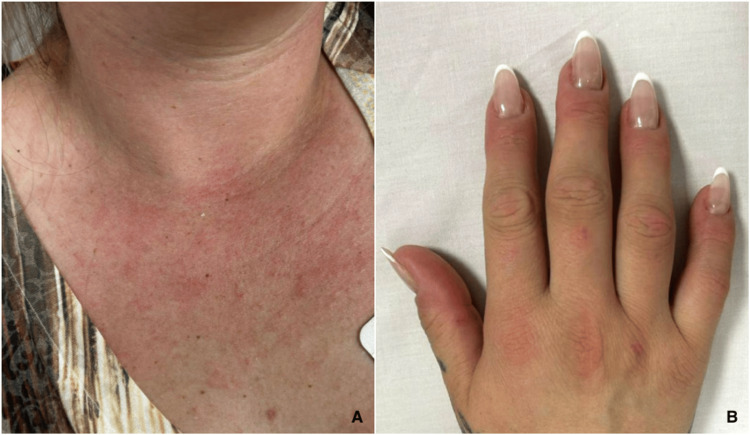
Cutaneous manifestations of anti-MDA5 dermatomyositis. (A) Macular erythematous rash over the anterior neck and chest, consistent with a V-sign pattern. (B) Gottron papules over the dorsal aspect of the hand. MDA5: melanoma differentiation-associated gene 5

At the time of admission, the patient complained of shortness of breath and exhibited signs of respiratory distress, with pulse oximetry readings ranging from 85% to 95%. Chest computed tomography (CT) revealed multifocal irregular opacities in a pattern consistent with organizing pneumonia (Figure 2A). Initial laboratory evaluation showed moderate lymphopenia, elevated lactate dehydrogenase (LDH), and elevated aspartate aminotransferase, while the initial autoimmune panel was negative, including rheumatoid factor, antinuclear, anti-Sjögren’s-syndrome-related antigen A (anti-Ro/SSA), anti-Sjögren’s-syndrome-related antigen B (anti-La/SSB), anti-Smith (anti-Sm), anti-ribonucleoprotein (anti-RNP), and anti-histidyl-transfer ribonucleic acid synthetase (anti-Jo1) antibodies (Table 1). Serum ferritin was not measured during the initial admission or readmission; therefore, it could not be incorporated into formal risk stratification.

**Figure 2:**
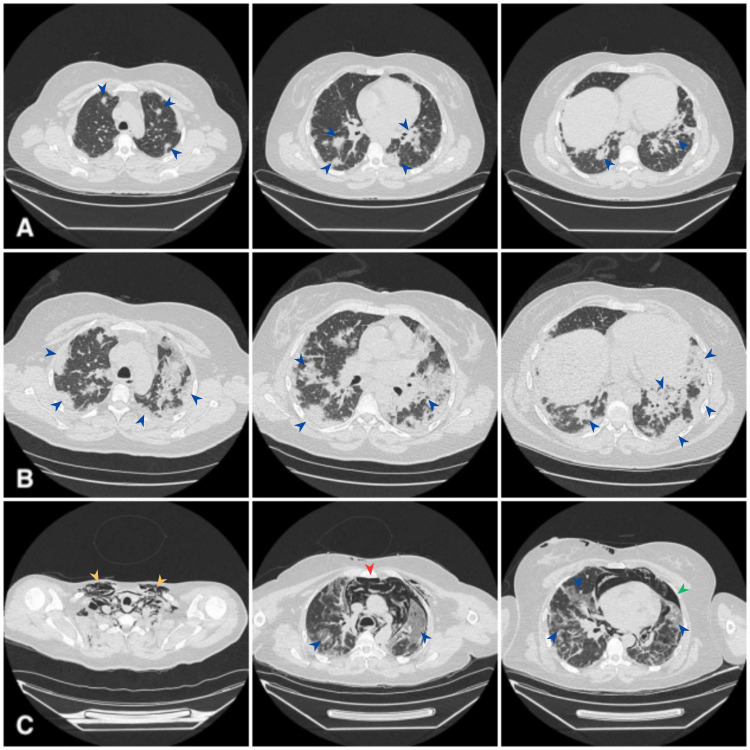
Chest computed tomography (CT) findings during disease progression. (A) Chest CT at admission showing bilateral multifocal irregular opacities with an organizing pneumonia pattern (blue arrows). (B) Follow-up chest CT after treatment initiation demonstrating worsening diffuse ground-glass opacities and the progression of pulmonary involvement (blue arrows). (C) Chest CT at readmission showing the deterioration of pulmonary lesions (blue arrows), extensive subcutaneous emphysema (yellow arrows), pneumomediastinum (red arrow), and a small left-sided pneumothorax (green arrow).

**Table 1:** Laboratory findings during the disease course. IU/mL, international units per milliliter; U/L, units per liter; mm³, cubic millimeter; anti-Ro/SSA, anti-Sjögren’s-syndrome-related antigen A antibody; anti-La/SSB, anti-Sjögren’s-syndrome-related antigen B antibody; anti-Sm, anti-Smith antibody; anti-RNP, anti-ribonucleoprotein antibody; anti-Jo1, anti-histidyl-transfer ribonucleic acid synthetase antibody; anti-MDA5, anti-melanoma differentiation-associated gene 5 antibody

Laboratory parameter	At initial admission	At readmission	Reference range
Lymphocyte count	851/mm³	246/mm³	1000-5000/mm³
Lactate dehydrogenase	454 U/L	359-409 U/L	<247 U/L
Aspartate aminotransferase	323 U/L	103-141 U/L	<35 U/L
Rheumatoid factor	<10 IU/mL	Not repeated	<14 IU/mL
Antinuclear antibody	Non-reactive	Not repeated	Non-reactive
Anti-Ro/SSA antibody	<0.4 U/mL	Not repeated	<7 U/mL
Anti-La/SSB antibody	<0.4 U/mL	Not repeated	<7 U/mL
Anti-Sm antibody	<0.8 U/mL	Not repeated	<7 U/mL
Anti-RNP antibody	1.4 U/mL	Not repeated	<7 U/mL
Anti-Jo1 antibody	<0.3 U/mL	Not repeated	<7 U/mL
Anti-MDA5 antibody	Pending at admission	Positive	Negative

An infectious workup was performed before immunosuppressive therapy was initiated. Blood cultures showed no growth, serum procalcitonin was low at 0.06 ng/mL (reference value: <0.5 ng/mL), and C-reactive protein was elevated at 19 mg/L (reference value: <5 mg/L). The elevated C-reactive protein level was considered compatible with the underlying systemic inflammatory process and, in isolation, was not interpreted as evidence of active bacterial infection. Based on the clinical symptoms, additional laboratory and imaging findings, and the previous lack of improvement despite broad-spectrum antibiotic therapy, the working diagnosis at that time was dermatomyositis-associated interstitial lung disease, with concern for a rapidly progressive phenotype. In view of the absence of microbiological evidence of bloodstream infection and the strong suspicion of an inflammatory autoimmune process, aggressive immunosuppression was initiated with intravenous methylprednisolone pulse therapy (500 mg once daily for three consecutive days), followed by oral prednisone 60 mg/day (0.86 mg/kg/day for a body weight of 70 kg), given the severity of the pulmonary involvement. Prednisone was administered in combination with tacrolimus (5 mg twice daily) and mycophenolate mofetil, which was initiated at 500 mg twice daily with planned dose escalation to 3 g/day rather than immediate full-dose therapy, aiming to improve gastrointestinal and hematologic tolerability in the setting of severe systemic illness and close laboratory monitoring.

There was an initial partial response with improvement in joint and skin manifestations with the initial treatment. However, respiratory compromise persisted, the CT findings worsened (Figure 2B), and transfer to the intensive care unit (ICU) was necessary. Adjuvant therapy with intravenous immunoglobulin (IVIg) at a dose of 400 mg/kg/day for five days was then prescribed, which led to significant improvement. The patient was discharged from the hospital approximately one month after admission, maintaining immunosuppressive combined therapy with prednisone and mycophenolate mofetil, and scheduled for follow-up appointments with the pulmonology and rheumatology teams.

Five days after discharge, the patient returned to the hospital with acute respiratory failure following an intense coughing fit the previous night, presenting with extensive cervical subcutaneous emphysema. A chest CT scan (Figure 2C) revealed a significant deterioration of pulmonary lesions, as well as a voluminous pneumomediastinum and a minor left-sided pneumothorax. Laboratory tests also worsened, with severe lymphopenia and persistently elevated tissue injury markers (Table 1). The patient was treated in the emergency room and readmitted to the ICU.

Due to refractory disease progression, rescue therapy was initiated in consultation with the rheumatology team and consisted of a new pulse of methylprednisolone 500 mg/day for three consecutive days, a second cycle of IVIg at the same dose as previously administered, the reintroduction of tacrolimus, and a single intravenous cyclophosphamide pulse of 1200 mg, a dose consistent with the recommended range of 500-1000 mg/m² for her estimated body surface area, based on a weight of 70 kg and height of 1.70 m. Sulfamethoxazole-trimethoprim was started for *Pneumocystis jirovecii* prophylaxis. Throughout hospitalization, the patient was closely monitored for opportunistic infections, with no clinical, laboratory, or microbiological evidence of infection.

During hospitalization, the myositis panel confirmed isolated anti-MDA5 positivity by immunoblot assay (Table 1). Despite lung-protective mechanical ventilation and hemodynamic support, the patient’s ICU course was complicated by progressive refractory hypoxemia and hemodynamic deterioration, culminating in a fatal outcome on the 10th day following readmission.

## Discussion

ILD is the predominant extramuscular manifestation in anti-MDA5 DM, although its prevalence varies according to ethnic and geographic differences. According to a review by Nombel et al., ILD affects between 80% and 100% of Asian cohorts, whereas the rates are lower in Western populations, ranging from 40% to 70%. The RP-ILD phenotype follows this pattern, with an incidence ranging from 39% to 71% in Asia and from 20% to 57% in Western populations [1]. Recent data from a meta-analysis by Yang et al. indicate that mortality is concentrated within the first six months after disease onset and may approach 50%, even among patients receiving intensive care [9].

An international retrospective cohort study comparing 34 Brazilian to 65 Japanese patients showed a significantly lower prevalence of ILD and RP-ILD among Brazilians (50.0% and 3.3%, respectively) than Japanese patients (98.5% and 52.3%, respectively), with the classic DM phenotype predominating in Brazilians. Despite the lower frequency of RP-ILD, mortality was similar (8.8% versus 13.8%) [8]. In this scenario, the presented case reinforces the need for early surveillance and intervention even in populations with a lower reported predisposition to RP-ILD.

Spontaneous pneumomediastinum is one of the most severe structural complications of ILD associated with anti-MDA5 DM. In Asian cohorts, pneumomediastinum has been reported in 13%-35% of patients, with mortality rates ranging from 40% to 60% [5,6]. In a Japanese study, its occurrence was linked to significantly higher 12-month mortality rates compared to patients without the condition (53.3% versus 4.0%), reinforcing its role as a marker of advanced lung disease, as observed in the present case, in which pneumomediastinum accompanied rapid respiratory deterioration and a fatal outcome [10].

In clinical practice, risk prediction models such as Ferritin, Lactate Dehydrogenase, Anti-MDA5 Antibody, Imaging Score, and Rapidly Progressive Interstitial Lung Disease (FLAIR) may help integrate laboratory, radiological, and clinical variables into survival estimates for patients with anti-MDA5 DM-associated ILD. This model incorporates anti-MDA5 antibody status and intensity, ferritin levels, LDH levels, high-resolution computed tomography findings, and the presence of RP-ILD, categorizing patients as low, medium, or high risk, which in the validation study corresponded to one-year survival rates of 100%, 92.3%, and 15%, respectively [7].

Although the FLAIR score was not formally calculated in the present case, several features observed early in the disease course, including hypoxemia, elevated LDH levels, increased transaminases, lymphopenia, and progressive tomographic abnormalities, have been associated with poor prognosis in previous studies [1,5-7,11]. The absence of serum ferritin measurement precluded the complete application of the FLAIR model, as ferritin is one of its key components [7]. Nevertheless, the overall clinical, laboratory, and imaging findings already indicated an unfavorable prognosis. These findings support the use of structured risk stratification as an adjunctive tool to identify patients who may benefit from early intensive therapeutic strategies, particularly when clinical improvement is incomplete.

The therapeutic window for RP-ILD associated with anti-MDA5 DM is narrow, requiring early recognition and prompt aggressive immunosuppression. A step-up treatment strategy that starts with glucocorticoids alone and adds immunosuppressants as clinical deterioration occurs is associated with lower survival rates of only 28%-33% at six months, compared to 75%-89% with upfront combination therapy, also known as the “hit hard” strategy. The goal of the “hit hard” strategy is to control inflammation before irreversible alveolar damage progresses [1-4,6,11,12].

In the present case, combined immunosuppressive therapy was initiated before the confirmation of anti-MDA5 positivity, in accordance with recommendations for RP-ILD associated with idiopathic inflammatory myopathies. The initial regimen included intravenous methylprednisolone pulse therapy and tacrolimus, both considered first-line therapeutic options in this setting, together with adjunctive IVIg given the severity of the clinical presentation [2,6,12]. The selection of these agents was also influenced by their immediate availability at the treating hospital, allowing treatment to be initiated and intensified without delay. However, although consistent with the principle of the “hit hard” strategy, this regimen differed from the best-studied upfront triple-therapy protocol described by Tsuji et al., as intravenous cyclophosphamide was introduced only after relapse and structural complications had developed [3]. This delayed incorporation of cyclophosphamide, together with gradual mycophenolate titration, may have reduced the immediate immunosuppressive intensity compared with classic upfront triple therapy.

In refractory cases, rescue strategies such as Janus kinase inhibitors, including tofacitinib, therapeutic plasma exchange, and early referral for lung transplantation assessment, potentially with extracorporeal membrane oxygenation as a bridge to transplantation, have been explored [1-4,6]. However, these therapies were not readily available at the treating institution, limiting their timely use in the setting of rapidly progressive clinical deterioration. In this patient, the rapid progression of respiratory failure and the development of pneumomediastinum and pneumothorax ultimately precluded eligibility for lung transplantation.

The management of anti-MDA5 dermatomyositis associated with rapidly progressive interstitial lung disease remains challenging because of the lack of large randomized clinical trials. Consequently, therapeutic decisions are based primarily on cohort studies, case series, and expert consensus. Treatment strategies must balance the intensity of immunosuppression required to control rapidly progressive pulmonary inflammation against the increased risk of infection, underscoring the importance of *Pneumocystis jirovecii* pneumonia prophylaxis and close surveillance for opportunistic infections [2,6].

## Conclusions

The severe structural complications observed in this case, such as pneumomediastinum and pneumothorax, reinforce the heterogeneity of the anti-MDA5 disease phenotype and the lethality of RP-ILD. This report demonstrates that aggressive forms of the disease may occur even in populations with a lower reported prevalence of RP-ILD. These findings underscore the importance of early recognition, systematic risk stratification, and immediate intervention with intensive combined immunosuppression when signs of RP-ILD appear. Additionally, apparent early clinical improvement should not be interpreted as definitive disease control, as rapid relapse may occur.
